# Response to Barton et al. confirming that Burke et al. do not show a relationship between climate change and suicide

**DOI:** 10.1177/10398562241256801

**Published:** 2024-05-24

**Authors:** Andrew James Amos

**Affiliations:** 104397 Townsville, QLD

Dear Editor,

Judging by their response to my article on the lack of evidence of a relationship between climate change and suicide,^1^ Barton et al.^2^ have simply not understood it, as they repeat the key finding unchanged, without awareness.

They state my analysis was methodologically inappropriate because it reduced “the information contained in the data by aggregating across years and states” and “homogenises data across spectra that are obviously heterogeneous.” They prefer Burke et al.’s^3^ analysis because “it can detect fluctuations that almost certainly occur within years and states.”

Of course, the latter quote repeats the main point of my article. Burke et al.’s^3^ analysis is designed to detect fluctuations WITHIN YEARS (months, in fact) and WITHIN STATES (suburbs, in fact). Climate change occurs over many years and over the entire globe. Burke et al.^3^ take an association between variations in suicide and temperature over months within suburbs and implausibly assume it can be extrapolated to the level of years and countries.

As my article demonstrates, this is simply the wrong level of analysis. To illustrate by analogy, Burke et al.^3^ and Barton et al.^2^ appear to believe that if an association can be found between monthly temperature and the average height of waves for small patches of ocean, it can be assumed that there will also be an association between global temperature over decades and the absolute height of the world’s oceans. However, waves wax and wane around a mid-point. A higher wave comprises both a higher peak and a correspondingly lower trough, leaving the average height unchanged. An association between temperature and wave height tells you nothing about ocean height.

Similarly, Burke et al.^3^ show an association between variation in temperature and variation around some average level of suicide over short periods of time over small areas of land. They do not show an association between variation in temperature and variation in average suicide rates over long periods of time for large regions. By contrast with Burke et al.,^3^ my article targets the level of analysis necessary to reach conclusions about climate change: does variation in average temperature across large regions affect variation in average suicide rates across years? And answers no, it doesn’t.

Barton et al.^2^ suggest that the sheer number of references in the Lancet’s Countdown report^4^ is persuasive, neatly illustrating the second technique for misrepresenting scientific evidence described in my article, which is to “combine individually unconvincing studies to make claims about the importance of the larger set. … [The] agglomeration of disparate studies distracts attention from the fact that the suicide studies are not designed to consider climate change, and from the fact that none of the other studies establish strong evidence for a relationship between climate change and mental health outcomes either.”

I recommend that readers consider these self-evident limitations in Barton et al.’s^2^ analysis of my paper when deciding what weight to attribute to their assertions about the relationship between climate change and mental health.

## ORCID iD

Andrew James Amos https://orcid.org/0000-0002-9145-0212
